# Neglected Functions of TFCP2/TFCP2L1/UBP1 Transcription Factors May Offer Valuable Insights into Their Mechanisms of Action

**DOI:** 10.3390/ijms19102852

**Published:** 2018-09-20

**Authors:** Agnieszka Taracha, Grzegorz Kotarba, Tomasz Wilanowski

**Affiliations:** Laboratory of Signal Transduction, Nencki Institute of Experimental Biology of Polish Academy of Sciences, 3 Pasteur St., 02-093 Warsaw, Poland; a.taracha@nencki.gov.pl (A.T.); g.kotarba@nencki.gov.pl (G.K.)

**Keywords:** development, reproduction, brain disorders, Grainyhead

## Abstract

In recent years, the TFCP2 (transcription factor cellular promoter 2)/TFCP2L1 (TFCP2-like 1)/UBP1 (upstream binding protein 1) subfamily of transcription factors has been attracting increasing attention in the scientific community. These factors are very important in cancer, Alzheimer’s disease, and other human conditions, and they can be attractive targets for drug development. However, the interpretation of experimental results is complicated, as in principle, any of these factors could substitute for the lack of another. Thus, studying their hitherto little known functions should enhance our understanding of mechanisms of their functioning, and analogous mechanisms might govern their functioning in medically relevant contexts. For example, there are numerous parallels between placental development and cancer growth; therefore, investigating the roles of TFCP2, TFCP2L1, and UBP1 in the placenta may help us better understand their functioning in cancer, as is evidenced by the studies of various other proteins and pathways. Our review article aims to call the attention of the scientific community to these neglected functions, and encourage further research in this field. Here, we present a systematic review of current knowledge of the TFCP2/TFCP2L1/UBP1 subfamily in reproduction, embryonic development, renal function, blood-pressure regulation, brain function, and other processes, where their involvement has not been studied much until now.

## 1. Introduction

TFCP2 (transcription factor cellular promoter 2), TFCP2L1 (TFCP2-like 1) and UBP1 (upstream binding protein 1) constitute a distinct subfamily of Grainyhead-like (GRHL) transcription factors [1]. They are important for the regulation of the cell cycle, hematopoiesis, expression of the human immunodeficiency virus (HIV) genes, development of cancer, and Alzheimer’s disease. The above functions have been analyzed in detail in recent review articles [2,3,4], so we do not discuss them here. However, many underlying mechanisms are still unclear, and the interpretation of data is complicated by many issues, including redundancies between these proteins. For these reasons, our review aims to explore the less-known functions of TFCP2, TFCP2L1, and UBP1, because analysis of these functions may assist us in unraveling the general molecular mechanisms of action of these factors. This could not only improve our understanding of basic biological phenomena, but may also assist in the development of novel medical approaches to the treatment of various cancers, hematologic disorders, and other diseases.

In addition, we would like to recognize the emerging role of these proteins in animal production. At present, the significance of the transcription factors from the TFCP2/TFCP2L1/UBP1 subfamily in agriculture is not adequately appreciated. Here, we summarize increasing evidence that these proteins are involved in various aspects of animal breeding: prolificacy of sheep breeds, litter size in pigs, bovine reproduction, and response to heat stress in cattle. A more comprehensive effort to investigate the roles of TFCP2, TFCP2L1, and UBP1 in animal production is thus encouraged, as it may find practical applications in agriculture.

Although the Human Genome Organization (HUGO) Gene Nomenclature Committee approved the symbols TFCP2, TFCP2L1, and UBP1, many synonyms are still being used in the scientific literature. In our article, we strictly follow the HUGO nomenclature, but for the benefit of our readers we list these synonyms below:

TFCP2: CP2, TFCP2C, LSF (late SV40 factor), LBP-1c, LBP-1d

TFCP2L1: CRTR-1, LBP-9

UBP1: LBP-1a, LBP-1b, NF2d9.

## 2. Reproduction

Transcription factors from the TFCP2/TFCP2L1/UBP1 subfamily are involved in various processes related to reproduction. In gonads and adrenals, many *trans*-acting factors regulate the expression of the *CYP11A1* gene, which encodes mitochondrial cholesterol side-chain cleavage enzyme P450scc, a key enzyme catalyzing the conversion of cholesterol to pregnenolone. While steroidogenic factor 1 (SF-1) plays a key role in adrenals and testes, UBP1, under the regulation by its dimer partner TFCP2L1, can replace SF-1 function and activate the *CYP11A1* promoter in placental JEG-3 cells [5]. Reprogramming of the human germline, including global DNA demethylation, chromatin reorganization, and X-chromosome reactivation, is critical for development. Human primordial germ cells (hPGCs) express pluripotency genes, including kruppel-like factor 4 (KLF4) and TFCP2L1 [6]. These two factors, together with SRY box-17 (SOX17) and PR domain zinc finger protein 1 (BLIMP1), drive epigenome resetting and global hypomethylation in these cells [6].

Regarding the production of female gametes, several studies have demonstrated that miRNAs play important roles in folliculogenesis, a complex process where primordial follicles develop into preovulatory follicles, which leads to ovulation and to the release of mature oocytes. Chromatin immunoprecipitation (ChIP) and electrophoretic mobility shift assays (EMSA) revealed that transcription factor TFCP2 binds to the promoter region of miR-144 in murine granulosa cells (mGCs) [7]. Moreover, TFCP2 upregulates miR-144/451, which results in the inhibition of COX-2 expression and PGE2 production.

TFCP2 is also involved in the production of male gametes. The localization and expression profiles of yin yang 1 (YY1) and TFCP2 in mouse gonocytes and germ cells were investigated using immunohistochemistry and immunofluorescence [8]. It was found that YY1 is colocalized heterogeneously with TFCP2 to permit spermatogenesis. TFCP2 is also important for the commitment of spermatogonia, and for the progression of spermatogonia to spermatids. However, the interplay between these two transcription factors in maintaining stemness of spermatogonia and spermatogenesis is complex and not fully understood [8].

Pregnancy requires the proper functioning of TFCP2 and TFCP2L1. Physiological adaptations in the female organism are crucial for a successful pregnancy. A recent study conducted on rats discovered maternal thyroid enlargement by 18% in late pregnancy [9]. Molecular basis of this process was investigated using an RNA-seq approach, which identified 615 differentially expressed genes; 90.7% of these genes were upregulated in late pregnancy. Examination of the proximal promoter regions of selected upregulated genes revealed MYC-associated zinc finger protein (MAZ) and TFCP2 as two pivotal transcription factors responsible for the regulation of transcriptional activity of these genes [9]. Moreover, the sequence alignment (with Lasagne-Search 2.0, Cluster Bluster, and MatInspector software) of pregnancy-associated glycoprotein family (PAGs) pPAG2-L promoter in pigs detected a TFCP2 binding site, which may emphasize a regulatory role of this factor in mammalian reproduction [10]. The study on LH-β (luteinizing hormone β subunit) promoter activity among three breeds of Moroccan sheep, D’man, Sardi, and Timahdite, showed higher promoter activity in D’man compared to the other breeds [11]. In silico analysis of a 541 bp region of D’man LH-β promoter revealed binding sites for a number of transcription factors, including GATA-1/GATA-2, E4/th1, c-Ets, and TFCP2, which may explain the high prolificacy of this breed [11]. However, the authors did not demonstrate that TFCP2 actually binds to this site, so the links between this transcription factor and sheep prolificacy are not yet proven. Additionally, TFCP2L1, another member of the TFCP2/Grainyhead family, affects the enhancement of litter size in purebred Landrace female pigs, where, together with signal transducer and activator of transcription 2 (STAT2) and myogenic factor 6 (MYF6), it remains a key transcription factor influencing sow lifetime productivity (SLP) [12].

## 3. Embryonic Development

Regulation of expression of the male sex-determining region Y gene (SRY) is one of the crucial processes that control mammalian sex determination. SRY is a hybrid gene of DiGeorge syndrome critical region gene 8 (DGCR8) and SRY-Box 3 (SOX3), and TFCP2 binding motif is present in both [13]. EMSA confirmed that TFCP2 is a regulator of SRY gene transcription by directly binding to its promoter. However, targeted disruption of *Tfcp2* did not induce aberrant proportions of males and females in mice [14]. In the same study, using the yeast two-hybrid assay system, it was found that UBP1 could potentially compensate for the loss of TFCP2 by forming complexes with previously defined heteromeric partners of this transcription factor [14]. Interestingly, analysis of 11 polymorphisms in three candidate genes, including *Ubp1* in dogs, found no association with a canine disorder of sexual development (DSD) [15]. Thus, any links between TFCP2 and UBP1 transcription factors and sex determination in mammals remain highly questionable.

Studies in mice revealed that TFCP2L1 coordinates the development of two types of epithelial cells, intercalated (IC) and principal (PC) cells, in kidney collecting ducts [16]. TFCP2L1 turned out to be a critical regulator of IC–PC patterning, acting cell-autonomously in ICs, and non-cell-autonomously in PCs. In particular, it induces the expression of IC specific genes, including the proton-pumping ATPase (H^+^-ATPase) subunits and jagged 1 (*Jag1*), the latter being responsible for initiating Notch signaling in PCs and its inhibition in ICs. *Tfcp2l1* inactivation brings about the deletion of ICs, whereas inactivation of *Jag1* results in the loss of discrete IC and PC identities. In fact, TFCP2L1 plays a key role in the diversification of cell types and distinguishes the collecting duct from all other nephron segments [16].

TFCP2L1 also contributes to the differentiation of salivary-gland duct cells [17]. In *Tfcp2l1*-deficient mice, the expression of genes that are involved in duct maturation was reduced both in kidneys and in the salivary gland. Furthermore, these mice are characterized by an abnormal composition of secreted saliva and urine [18]. In contrast, cultured primary salivary human stem/progenitor cells (hS/PCs) display reduced mRNA levels of differentiated marker genes, including *TFCP21* [19]. A study on branching morphogenesis showed that, in the development of the mouse submandibular gland (SMG), in endbud cells, a heparan sulfate (HS)-binding growth factor, fibroblast growth factor 10 (FGF10) inhibits the expression of TFCP2L1 via the repression of Wnt5b ligand and noncanonical Wnt signaling [20,21]. Interestingly, in *Drosophila melanogaster*, the first discovered member of the TFCP2/Grainyhead transcription factor family—Grainyhead (GRH)—is involved in the development of trachea, where it regulates apical membrane growth and tube elongation under the control of Branchless/FGF signaling [22]. These findings suggest that this molecular mechanism controlling branching development is conserved from fly to man.

In addition, TFCP2L1 plays a key role in maintaining mouse embryonic stem-cell (mESC) pluripotency via the LIF/STAT3 signaling pathway [23]. Importantly, in the inner cell mass (ICM) of bovine blastocysts, chemical inhibition of JAK/STAT3 signaling repressed *Tfcp2l1* transcription, which suggests that the role of this signaling pathway in pluripotency regulation is conserved between mice and cattle [24]. UBP1 and TFCP2L1 are highly expressed in the human placenta and directly modulate the expression of genes coding for cytochrome P450scc, steroidogenic factor-1 (SF-1), and another member of the TFCP2/Grainyhead family, Grainyhead-like 1 (GRHL1, also known as LBP-32) [25,26,27]. Furthermore, the deletion of *Ubp1* in mice resulted in a defective yolk sac and placental vasculature with concomitant decrease in the thickness of the labyrinthine layer [28]. The above findings indicate that UBP1, TFCP2L1, and GRHL1 play a critical role in placenta development. It should be noted that the other two Grainyhead-like transcription factors, GRHL2 and GRHL3, are also highly expressed in the placenta, where GRHL2 is involved in the regulation of placental morphogenesis [29,30,31]. Thus, placenta seems to be the most important organ where all the members of the TFCP2/Grainyhead family of transcription factors are highly expressed, which is why future studies of their function in the placenta may yield very valuable information about these transcription factors.

## 4. Renal Function and Blood-Pressure Regulation

TFCP2, TFCP2L1, and GRHL1 transcription factors have been detected in the rat inner-medullary collecting ducts [32]. Computational analysis of conserved transcription factor binding sites identified a potential TFCP2 and/or TFCP2L1 binding site in a conserved region of the first intron of the aquaporin-2 (*Aqp2*) gene. It may suggest that one or both of these factors could play a role in the regulation of the water channel aquaporin-2 in the kidney [32].

As shown in a mouse model, TFCP2L1 is required for proper electrolyte excretion in the kidneys, and this function is essential for blood-pressure regulation [18]. UBP1, another member of the same subfamily, has been identified as a critical blood-pressure determinant in humans [33]. UBP1 is involved in cholesterol and steroid metabolism via the transcriptional activation of *CYP11A1*, the gene encoding the rate-limiting enzyme in steroidogenesis, cytochrome P450scc [26,34,35,36]. Therefore, UBP1 and its functional partners are considered to be components of a network controlling blood pressure [33]. These findings are particularly interesting, as another member of the TFCP2/Grainyhead family also plays an important role in blood-pressure regulation. The effects of loss of the *Grhl1* gene for gene expression in the kidneys, regulation of blood pressure, and heart rate in mice are well-documented [37,38]. For these reasons, future research into the involvement of transcription factors from the TFCP2/TFCP2L1/UBP1 subfamily in blood-pressure regulation is warranted, as it is likely to yield novel and interesting scientific as well as medically relevant findings.

In the Xenopus larval skin, *ubp1* is a target of regulation by Notch activity [39]. It is expressed primarily in beta-intercalating nonciliated cells (β-INCs) and it suppresses the ability of forkhead box I1 (FOXI1) to activate the expression of anion exchanger 1 (ae1) ectopically and, when misexpressed in embryos, the formation of alpha-INCs, promoting at the same time the formation of beta-INCs. Furthermore, UBP1 appears to execute a complete switch in subtype identity of proton-secreting cells (PSCs), by not only repressing the expression of *ae1*, but also by inducing *pendrin* and causing the vacuolar-type H^+^-transporting ATPase (H^+^v-ATPase) to localize basolaterally rather than apically. Interestingly, inhibition of *ubp1* activity using morpholinos did not confirm these observations, which might be due to the fact that UBP1 overlaps in function with related members of the Grainyhead family, TFCP2 and TFCP2L1, both of which are also expressed in the skin [39]. As has already been suggested in the literature, members of the TFCP2/TFCP2L1/UBP1 subfamily are known to heterodimerize and probably have overlapping functions [31,40,41,42,43].

The importance of TFCP2L1 in kidney development has already been discussed in an earlier chapter on embryonic development.

## 5. Brain Function

TFCP2 binds the acid-sensing ion channel 2a (ASIC2a) gene’s core promoter segment and thereby regulates *ASIC2a* expression that affects susceptibility to epilepsy [44]. In PC12 cells, glucose deficiency resulted in TFCP2 downregulating *ASIC2a*. On the other hand, hippocampal glucose hypometabolism, both in human patients with epilepsy and in rat epilepsy-model brains, increases *ASIC2a* expression by suppressing *TFCP2* expression, which further enhances the intrinsic excitability of CA1 pyramidal neurons and escalates seizure susceptibility in patients with temporal lobe epilepsy [44].

TFCP2 is also associated with progressive supranuclear palsy (PSP), the second most common form of parkinsonism after Parkinson’s disease [45]. PSP risk is linked to the H1B tau gene haplotype and can be explained by one SNP, htSNP167, which creates a TFCP2 binding site in the regulatory region of this gene [45].

Moreover, TFCP2 seems to be an important factor in major depressive disorder (MDD) [46]. Considering cAMP responsive element binding protein 1 (*CREB1*) (a sex-limited susceptibility gene for unipolar mood disorders), G to A transition at position –656 in its promoter creates a perfect match to the core of the TFCP2 binding site, reflecting a gain of function that is consistent with the dominant effect (penetrance ≥82%) of this variant on the development of depressive disorders among female heterozygous carriers [46,47]. This transition also has functional consequences for *CREB1* promoter activity in CATH.a neuronal cells. TFCP2 appears to regulate glycogen synthase kinase 3β expression [48] that has been implicated in the pathophysiology of both mood disorders and Alzheimer’s disease (AD) [49,50]. In addition, a noncoding polymorphism in the 3′ untranslated region of *TFCP2* has been reported to affect the risk of MDD and AD [51,52].

## 6. Other Functions

The expression of the *TFCP2* gene is highly upregulated in regenerating cartilage tissue in a rabbit femoral groove model [53]. The authors speculate that this is due to the fact that TFCP2 is involved in the development of mesenchymal stem cells, but they do not provide any evidence to support their claim. Interestingly, some other members of the TFCP2/Grainyhead family are involved in cutaneous wound healing—GRH in *Drosophila melanogaster* and GRHL3 in mice [54,55,56]. Further investigations into the molecular mechanisms of TFCP2 involvement in cartilage regeneration are thus warranted, as this could be yet another example of a TFCP2/Grainyhead family member participating in a regenerative process.

TFCP2 might be linked to the response to heat stress in cattle [57]. A TFCP2-binding site was found in the 5′ untranslated region (UTR) of a gene coding for heat-shock protein 70.1 (*Hsp70.1*). Furthermore, the sequence of this binding site is altered in riverine buffalo (*Bubalus bubalis*), in comparison with taurine cattle (*Bos taurus*), and these species display different responses to heat-stress conditions. Unfortunately, this study was not followed up by any more detailed molecular analyses, which is why the role of TFCP2 in response to heat stress remains speculative. An additional concern is that TFCP2, TFCP2L1. and UBP1 bind to the same consensus DNA sequence [58]. For this reason, without further studies, it is not possible to determine which of these factors actually bind(s) to the *Hsp70.1* promoter in cattle.

UBP1 may be important for the regulation of drug metabolism [59]. It binds to a positive regulatory element for xenobiotic response element (PREX) in the *Cyp2a8* gene. This study was carried out using Syrian hamster liver extracts. Furthermore, ectopic expression of UBP1 increases the transcriptional output of *Cyp2a8* promoter [59]. These results are consistent with earlier studies, which demonstrated that UBP1 regulates the expression of *Cyp2d9* in murine liver [60]. There are no data regarding the involvement of TFCP2 and TFCP2L1 in xenobiotic response and drug metabolism. However, it is known that TFCP2L1 and UBP1 regulate the expression of another member of the cytochrome P450 superfamily of enzymes, CYP11A1, in the placenta [26,27]. Clearly, there is a need to further investigate the relationship between TFCP2/Grainyhead transcription factors and cytochrome P450 enzymes, as this may shed new light on the functioning of this family of transcription factors.

Finally, TFCP2 has been shown to play a central regulatory role in responses to therapeutic interferon in patients with multiple sclerosis [61]. Polymorphisms in the *TFCP2L1* gene are also associated with susceptibility to various forms of the West Nile virus (WNV) neuroinvasive disease at genome-wide significance thresholds [62]. A variant (rs17006292) in *TFCP2L1* has been associated with Behcet’s disease among Han Chinese people [63].

**Figure 1:**
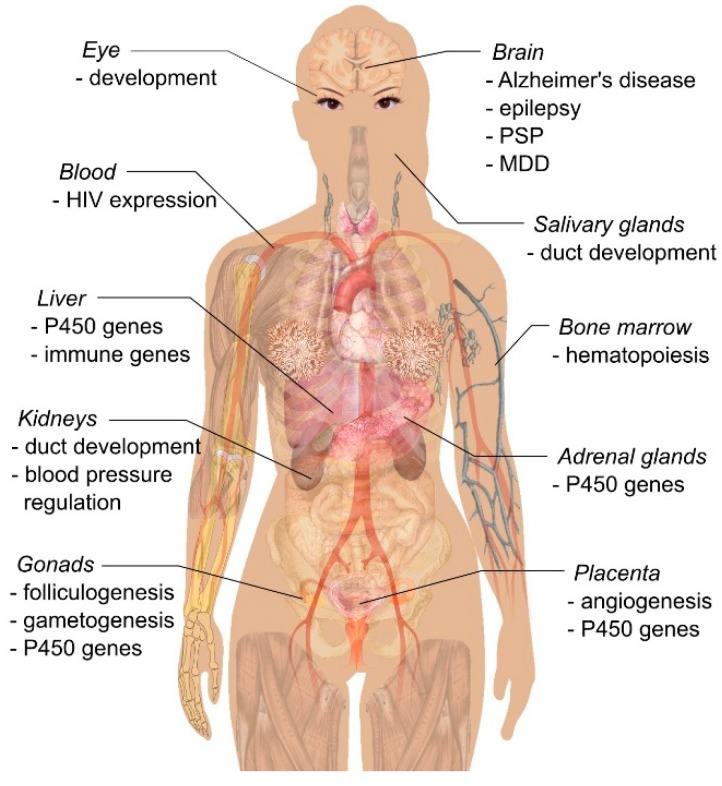
Functions of TFCP2 (transcription factor cellular promoter 2), TFCP2L1 (TFCP2-like 1), and UBP1 (upstream binding protein 1) in various organs. The only proven disease links or targets of regulation are shown. Note that, although TFCP2 and UBP1 are expressed ubiquitously, their role in most tissue and organs remains unknown. The functions of these factors in the liver, bone marrow, eye development, and regulation of HIV expression were summarized in an earlier review [3], therefore we do not discuss them here. Background image source: Wikimedia Commons.

**Figure 2:**
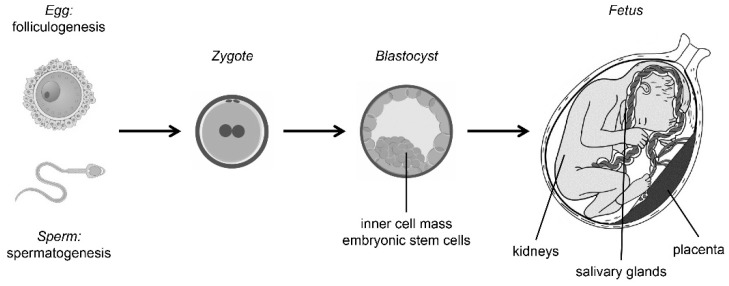
Developmental functions of TFCP2/TFCP2L1/UBP1 transcription factors.

The known functions of TFCP2/TFCP2L1/UBP1 transcription factors are summarized in Figure 1 and Figure 2.

## 7. Conclusions and Future Perspectives

Transcription factors from the TFCP2/TFCP2L1/UBP1 subfamily fulfil very diverse and wide-ranging roles in mammals and other animals. However, in most cases, available information is scarce, and even in the best studied examples some important questions remain unanswered. Redundancy is one of the key complicating issues, as these proteins display functional redundancy in multiple contexts [14,28,42]. This redundancy can be explained at the molecular level: TFCP2, TFCP2L1, and UBP1 can bind to the same DNA sequences [58], they share very high degrees of protein-sequence homology, and they have very similar domain structures [64]. For these reasons, the interpretation of experimental results is complicated as, in principle, any one of these factors may substitute for the lack of another [42]. Investigating redundant systems is a challenging task. One possible approach could be to overexpress one transcription factor in an ectopic environment in which the other family members are not expressed, and investigate its occupancy at target sites using ChIP followed by next-generation sequencing, as was recently did in the case of FOXA2 [65]. Unfortunately, TFCP2 and UBP1 are ubiquitously expressed in developing and adult mice, as well as in all human cell lines examined to date [3], so finding an appropriate ectopic environment could be very difficult. Other approaches are provided by various in silico methods, such as networks, graph theory, and systems theory [66]. Using tissue-culture cells with double knockdown of two redundant genes, or mouse strains harboring a combination of a standard knockout of one gene and a conditional knockout of a second gene, has also been proposed to resolve problems associated with redundancy [3]. Further help in dissecting their redundant roles might come from analyzing their interactions with other proteins, and their mutually exclusive involvement in protein complexes [67].

Nevertheless, it is necessary to continue research efforts, as these factors are very important in cancer, Alzheimer’s disease, and other human conditions [3,4]. They can be attractive targets for drug development, especially since nowadays the activity of transcription factors can be directly modulated using small molecule compounds [68]. Furthermore, there are numerous reports indicating that knowledge about TFCP2, TFCP2L1, and UBP1 might also prove valuable in the field of agriculture, in animal production. Thus, studying their roles in reproduction, embryonic development, and other processes highlighted here should enhance our understanding of mechanisms of functioning of these transcription factors, and the same mechanisms may govern their functioning in medically and agriculturally relevant contexts. For example, there are numerous parallels between placental development and cancer growth [69], and angiogenesis and Notch signaling are essential for both of these processes [70,71]. Interestingly, transcription factors TFCP2 and UBP1 are both important in angiogenesis; furthermore, Notch signaling regulates the expression of TFCP2 and UBP1 in hepatocellular carcinoma and in the Xenopus larval skin, respectively, while TFCP2L1 indirectly drives Notch signaling in the kidney collecting-duct system (recently reviewed in Reference [4] and in this article). For these reasons, investigating the roles of TFCP2, TFCP2L1, and UBP1 in the placenta may help us better understand their functioning in cancer, as is evidenced by studies of various other proteins and pathways [69].

## Abbreviations

AD	Alzheimer’s disease
AE1	anion exchanger 1
AQP2	aquaporin-2
ASIC2a	acid-sensing ion channel 2a
ChIP	chromatin immunoprecipitation
COX	cyclooxygenase
CREB1	cAMP responsive element binding protein 1
CYP	cytochrome P450
DGCR8	DiGeorge syndrome critical region gene 8
DSD	disorder of sexual development
EMSA	electrophoretic mobility shift assay
FGF	fibroblast growth factor
GRH	Grainyhead
GRHL	Grainyhead-like
H^+^-ATPase	proton-pumping ATPase
H^+^v-ATPase	vacuolar-type H^+^-transporting ATPase
hPGC	human primordial germ cell
HS	heparan sulfate
HSP	heat shock protein
hS/PC	human stem/progenitor cell
IC	intercalated cell
ICM	inner cell mass
INC	intercalating nonciliated cell
JAK	Janus kinase
KLF	kruppel-like factor
LBP	leader-binding protein
LH-β	luteinizing hormone β subunit
LIF	leukemia inhibitory factor
LSF	late SV40 (simian virus 40) factor
MAZ	MYC-associated zinc finger protein
MDD	major depressive disorder
mESC	mouse embryonic stem cell
mGC	murine granulosa cell
MYF	myogenic factor
PAG	pregnancy-associated glycoprotein
PC	primordial cell
PGE	prostaglandin E
PREX	positive regulatory element for xenobiotic response element
PSC	proton-secreting cell
PSP	progressive supranuclear palsy
SF1	steroidogenic factor 1
SLP	sow lifetime productivity
SMG	submandibular gland
SNP	single nucleotide polymorphism
SOX	SRY-box
SRY	sex-determining region Y
STAT	signal transducer and activator of transcription
TFCP2	transcription factor cellular promoter-2
TFCP2L1	TFCP2-like 1
UBP1	upstream binding protein 1
UTR	untranslated region
WNV	West Nile virus
YY1	yin yang 1
